# Complex effects of childhood abuse, subjective social status, and trait anxiety on presenteeism in adult volunteers from the community

**DOI:** 10.3389/fpsyg.2022.1063637

**Published:** 2022-12-20

**Authors:** Rintaro Nibuya, Akiyoshi Shimura, Jiro Masuya, Yoshio Iwata, Ayaka Deguchi, Yoshitaka Ishii, Yu Tamada, Yota Fujimura, Hajime Tanabe, Takeshi Inoue

**Affiliations:** ^1^Department of Psychiatry, Tokyo Medical University, Tokyo, Japan; ^2^Fuji Psychosomatic Rehabilitation Institute Hospital, Fujinomiya, Shizuoka, Japan; ^3^Department of Psychiatry, Tokyo Medical University Hachioji Medical Center, Tokyo, Japan; ^4^Faculty of Humanities and Social Sciences, Shizuoka University, Shizuoka, Japan

**Keywords:** childhood abuse, Presenteeism, subjective social status, trait anxiety, path analysis

## Abstract

**Background:**

Presenteeism, which is reduced productivity levels owing to physical or mental problems, causes substantial economic loss. It is known to be associated with personal and working environment factors, but the mechanism has not been fully clarified to date. Therefore, we analyzed the effects of childhood abuse on presenteeism of general adult workers, and the mediating effects of subjective social status and trait anxiety.

**Methods:**

From 2017 to 2018, a cross-sectional survey was performed, and 469 adult workers provided written consent. Demographic information, and results from the Child Abuse and Trauma Scale, Subjective Social Status, State–Trait Anxiety Inventory form Y, and Work Limitations Questionnaire were investigated. Multiple linear regression and path analyzes were performed.

**Results:**

Childhood abuse indirectly affected current presenteeism *via* subjective social status and trait anxiety. Presenteeism was directly affected only by trait anxiety, childhood abuse directly affected subjective social status and trait anxiety, and subjective social status affected trait anxiety.

**Conclusion:**

This study clarified the long-term effects of childhood abuse on presenteeism in adulthood *via* trait anxiety. Therefore, assessing childhood abuse, subjective social status, and trait anxiety may help to elucidate the mechanism of workplace presenteeism and develop measures against it.

## Introduction

Whereas “absenteeism” means missing work due to physical and mental problems, “presenteeism” means a decreased ability to work due to health problems, despite attending work (Smith, 1970; Auren, 1995). Presenteeism is known to be one of the highest health-associated costs. Psychiatric disorders, including depression, are major causes of presenteeism (Collins et al., 2005; Nagata et al., 2018). The prevalence of mood disorders and the certification of workers’ accident compensation for mental disorders are increasing recently in some countries, particularly in Japan (Yamauchi et al., 2017), and thus, presenteeism due to mental health problems is predicted to further increase in the future. Therefore, improving the mental health situation in the workplace is an important issue in public health. Various stress factors have been reported as risk factors for presenteeism, such as job requirements, remote work, control, compensation, procedural fairness, interactive fairness, and support from colleagues/managers, etc., (Yang et al., 2015 2019; Oshio et al., 2017; Kinman, 2019; Taya et al., 2020; Hayashida et al., 2021; Shimura et al., 2021). However, not only occupational factors but also other personal factors may impair workers’ health and result in subsequent presenteeism. For example, sleep disturbance, circadian rhythm misalignment, depression, physical illness, mental and physical stress responses, and irregular diets are known to have substantial effects on presenteeism (Okumura and Higuchi, 2011; Kinman, 2019; Furuichi et al., 2020; Ishibashi and Shimura, 2020; Hayashida et al., 2021; Shimura et al., 2022). However, personal factors exacerbating presenteeism have not been fully elucidated to date.

Previous studies have demonstrated that childhood abuse, inadequate parenting, and adverse experiences are risk factors for developing depression (Kessler and Magee, 1993; Caspi et al., 2003; Toda et al., 2015; Infurna et al., 2016). For example, women with a history of childhood abuse have an increased risk of depression. Moreover, the severity of abuse and the severity of future depression have a linear association (Wise et al., 2001), and genetic vulnerabilities and adverse experiences, such as childhood abuse, interact and play a role in the development of anxiety and affective disorders (Schiele et al., 2016). On the other hand, it has also been suggested that low subjective social status (SSS) is associated with many types of mental dysfunction (Scott et al., 2014). The SSS measures people’s perceptions of where they are in the social hierarchy. It is well known that objective socioeconomic status (OSES), such as income, occupation, and educational background, affects health, and people with a lower OSES tend to have poorer health. In recent years, the association between SSS and health has attracted much attention. This is because it was demonstrated that even after controlling for the effects of OSES in multivariate analysis, SSS has a statistically significant effect on health that is equal to or greater than that of OSES (Adler et al., 2000; Ostrove et al., 2000; Singh-Manoux et al., 2003; Operario et al., 2004). The fact that SSS is associated with health, independently of the effects of OSES, suggests the existence of yet unidentified pathways and mechanisms. Furthermore, childhood abuse is known to increase the levels of trait anxiety (the tendency to be anxious owing to a person’s personality) in adulthood, resulting in increased physical health problems, insomnia, and anxiety disorders (Sandi and Richter-Levin, 2009; Uchida et al., 2018; Deguchi et al., 2021). As there is a long-time interval between stressful experiences in childhood and depressive symptoms in adulthood, it is assumed that there are some mediators linking them. Previous cross-sectional studies have reported the mediating roles of subjective social status and trait anxiety on the associations between the experience of abuse or inadequate parenting in childhood and depressive symptoms in adulthood (Uchida et al., 2018; Higashiyama et al., 2019; Deguchi et al., 2021; Hayashida et al., 2021). As mentioned above, depression and presenteeism are closely associated, and depression-induced presenteeism is a major social problem (Okumura and Higuchi, 2011; Nagata et al., 2018). Therefore, childhood abuse experiences may influence the presenteeism of adult workers through subjective social status and trait anxiety, with long-term effects.

Few studies to date have investigated the association between childhood abuse and presenteeism. It was reported that a history of childhood abuse affects current presenteeism, and the association is mediated by depression and anxiety (De Venter et al., 2020). Another study reported that experiences of childhood victimization from peers in school or the community affect current presenteeism through neuroticism and perceived job stressors (Hashimoto et al., 2022). Childhood victimization is not childhood abuse, but shares several common features in that both increase neuroticism and depression, and mediate the association between neuroticism and depression (Tachi et al., 2019; Masuya et al., 2022). However, to the best of our knowledge, there have been no studies to date on whether subjective social status and trait anxiety have mediating roles between the history of childhood abuse and current presenteeism.

Based on the above, we hypothesized that childhood abuse indirectly affects presenteeism through its impact on subjective social status and trait anxiety. By performing a questionnaire survey on adult workers, we evaluated childhood abuse, subjective social status, trait anxiety, and presenteeism, and analyzed the associations and mediations among these factors.

## Materials and methods

### Subjects

From 2017 to 2018, a cross-sectional survey using self-administered questionnaires was performed on 1,237 adult workers. Subjects were 20 years or older and the participation to this survey was voluntary. A total of 469 subjects (210 men and 259 women; mean age: 40.9 ± 11.9 years) provided written consent for the academic use of their data, and provided valid answers to the questionnaires that were used in the analysis. This study was approved by the Ethics Committee of Tokyo Medical University (study approval no.: SH3502) and was performed in accordance with the Declaration of World Medical Association Declaration of Helsinki Ethical principles for medical research involving human subjects (2013).

### Questionnaires

#### Work limitations questionnaire (WLQ) short form

The WLQ is a self-administered survey to measure health-associated working disability. Time demands, physical demands, mental interpersonal demands, and output demands are evaluated (Lerner et al., 2001). In this study, the validated Japanese short-form version was used (Takegami et al., 2014). The short form consists of 4 subitems, i.e., “time management (2 items),” “physical tasks (2 items),” “mental interpersonal tasks (2 items),” and “output tasks (2 items).” From the results of the percentage of time and frequency of failure of individuals to perform their duties owing to health problems in the previous 2 weeks, it is possible to calculate the percentage by which labor productivity has decreased owing to presenteeism relative to the original performance (% productivity loss). The WLQ calculates the percentage of productivity loss due to health problems in the previous 2 weeks. A higher %productivity loss indicates more difficulties in performing one’s job. WLQ has the most reliable correlation with actual work performance among the measuring methods of presenteeism (Gardner et al., 2016).

### State–trait anxiety inventory form Y (STAI-Y): Trait anxiety

The STAI-Y is a self-administered survey that assesses trait and state anxiety (Spielberger, 1983). Trait anxiety, which represents a relatively stable reaction tendency to anxiety-inducing experiences, was investigated in this study. The validated Japanese version of the STAI-Y was used (Hidano et al., 2000). This is a scale composed of 20 items that measures relatively stable reaction tendencies to anxiety experiences. Questions are answered on a 4-point scale ranging from “almost never (1 point)” to “almost always (4 points)”. The maximum score is 80, with higher scores indicating higher trait anxiety.

### Child abuse and trauma scale (CATS)

Childhood abuse was assessed by the CATS, which is a self-administered questionnaire (Sanders and Becker-Lausen, 1995). This questionnaire evaluates the family environment of the subjects as a child, and asks how the subjects felt about the treatment they received from their parents. The CATS consists of sexual abuse, neglect/negative home atmosphere, punishment, and others. The validated Japanese version of the CATS was used in this study (Tanabe et al., 2010). The Japanese version of the CATS consists of 38 items, divided into 3 subscales, namely, sexual abuse (6 items), neglect/negative home atmosphere (14 items), punishment (6 items), and others (12 items). A 4-point Likert scale was used for the evaluation of each item (0 = never, 1 = rarely, 2 = sometimes, 3 = very often, and 4 = always).

### Subjective social status (SSS)

The SSS questionnaire is a self-administered questionnaire that evaluates the participants’ perceptions of which social stratum they belong to. The questionnaire asks respondents the question “If this society was divided into 10 social strata, which stratum do you think you would belong to?” (1: lowest; 10: highest) (Adler et al., 2000; Scott et al., 2014).

### Statistical analysis

Covariance structure analysis with the robust maximum likelihood estimation method was performed using IBM SPSS Statistics Version 28 and Mplus 8.5 software (Muthén & Muthén). In this study, the comparative fit index (CFI) and root mean square error of approximation (RMSEA) were used to evaluate the goodness of fit of the model. A good fit was defined as a CFI > 0.97 and RMSEA < 0.05, and an acceptable fit was defined as CFI > 0.95 and RMSEA < 0.08 (Schermelleh-Engel et al., 2003). A *value of p* of less than 0.05 was considered to indicate a statistically significant difference between groups. SSS and trait anxiety are known to be mediators of the effect of childhood abuse on depressive symptoms (Uchida et al., 2018; Higashiyama et al., 2019; Deguchi et al., 2021). Therefore, an indirect effect, in which childhood abuse affects SSS and trait anxiety, and then these affect presenteeism, was assumed. Based on this hypothesis, a path model was created.

## Results

Table 1 shows the demographic characteristics and the results of measurements of the 469 adult workers. A lower SSS (*r* = −0.112, *p* = 0.015), past history of psychiatric disease (*p* = 0.019), current psychiatric disease (*p* < 0.001), STAI-Y trait anxiety score (*r* = 0.414, *p* < 0.001), and total score of the CATS (*r* = 0.317, *p* < 0.001) were associated with worse presenteeism. No other demographic factor was associated with presenteeism.

**Table 1:** Patient characteristics, STAI-Y, CATS, and SSS scores and their association with WLQ %productivity loss score.

Characteristic or measure	Value (number or mean ± SD)	Correlation (*r*) with or the difference of WLQ %productivity loss score (mean ± SD of WLQ %productivity loss score, *t*-test)
Age (years)	40.9 ± 11.9	*r* = −0.062, *p* = 0.178
Sex (men: women)	210: 259	Men (4.0% ± 4.3%) vs. women (4.3% ± 4.2%), *p* = 0.440 (*t*-test)
Education years	14.8 ± 1.78	*r* = −0.081, *p* = 0.081
SSS	5.14 ± 1.63	*r* = −0.112, *p* = 0.015
Past history of psychiatric disease (yes: no)	52: 417	Yes (5.5% ± 4.6%) vs. no (4.0 ± 4.2%), *p* = 0.019 (*t*-test)
Current psychiatric disease (yes: no)	19: 445	Yes (7.8% ± 5.0%) vs. no 4.0% ± 4.0%, *p* < 0.001 (*t*-test)
STAI-Y score (trait anxiety)	43.3 ± 10.4	*r* = 0.414, *p* < 0.001
CATS score	27.5 ± 20.7	*r* = 0.317, *p* < 0.001
WLQ %productivity loss score	4.1% ± 4.1%	−

STAI-Y, State–Trait Anxiety Inventory form Y; CATS, Child Abuse and Trauma Scale; SSS, Subjective Social Status (lowest: 1 to highest: 10); WLQ, Work Limitations Questionnaire

Table 2 shows the results of multiple regression analysis in which presenteeism was set as a dependent variable and the other factors as independent variables. Multicollinearity was tested using the VIF values, and it was determined that there was no obvious multicollinearity. STAI-Y trait anxiety score (standardized coefficient = 0.401, *p* < 0.001) and current psychiatric disease (0.152, *p* = 0.002) were identified as factors associated with presenteeism. The coefficient of determination (adjusted *R*^2^) of this model was 0.190.

**Table 2:** Results of multiple regression analysis with WLQ %productivity loss score as the dependent variable.

Independent variable	Beta	*p*	VIF
STAI-Y score (trait anxiety)	0.401	< 0.001	1.259
Current psychiatric disease	0.152	0.002	1.307
CATS total score	0.039	0.381	1.130
SSS	0.035	0.457	1.281
Sex	0.003	0.952	1.070
Past history of psychiatric disease	−0.059	0.230	1.361
Education years	−0.083	0.103	1.479
Age	−0.091	0.058	1.301
Adjusted *R*^2^ = 0.190, *F* = 14.6, *p* < 0.001			

Beta = standardized partial regression coefficient; VIF = variance inflation factor. STAI-Y, State–Trait Anxiety Inventory form Y; CATS, Child Abuse and Trauma Scale; SSS, Subjective Social Status; WLQ, Work Limitations Questionnaire. Independent variables: sex (male = 1, female = 2), age, education years, psychiatric history (no = 1, yes = 2), current psychiatric disease (no = 1, yes = 2), STAI-Y trait anxiety score, CATS score, SSS

**Figure 1:**
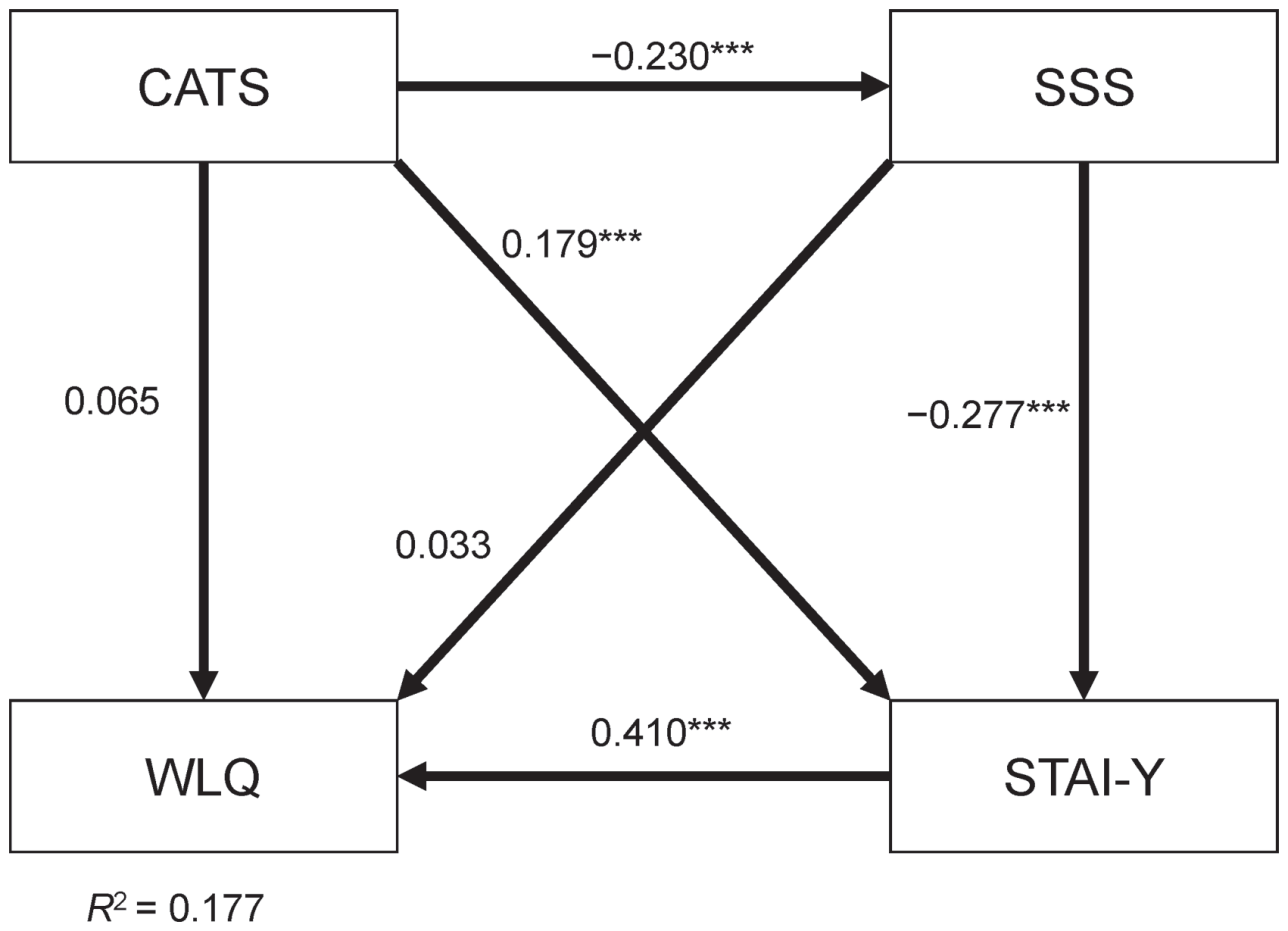
Results of covariance structure analysis using a path model, showing the scores of State–Trait Anxiety Inventory form Y (STAI-Y), Child Abuse and Trauma Scale (CATS), Subjective Social Status (SSS), and Work Limitations Questionnaire (WLQ) of 469 adult workers. The numbers beside the arrows indicate the direct standardized path coefficients. Indirect effects through the variables are explained in the Results section. ***p < 0.001.

Figure 1 shows the results of the path analysis with CATS score (childhood abuse), SSS score (subjective social status), STAI-Y score (trait anxiety), and WLQ score (presenteeism). This was a saturated path model. The CFI was 1.000 and the RMSEA was 0.000, indicating that the goodness of fit of the model was a good fit. Regarding significant direct effects, childhood abuse experience directly increased trait anxiety (standardized path coefficient = 0.179, *p* < 0.001) and directly decreased SSS (−0.230, *p* < 0.001). A higher SSS directly decreased trait anxiety (−0.277, *p* < 0.001), and trait anxiety directly worsened presenteeism (0.410, *p* < 0.001). Regarding indirect effects, childhood abuse experience had a significant indirect effect on trait anxiety through SSS (standardized indirect path coefficient = 0.064, *p* < 0.001). Childhood abuse experience also had a significant indirect effect on presenteeism through trait anxiety (0.073, *p* < 0.001). SSS had a significant indirect effect on presenteeism through trait anxiety (−0.113, *p* < 0.001). Childhood abuse experience had a significant indirect effect on presenteeism through paths including both SSS and trait anxiety (0.026, *p* < 0.001): trait anxiety mediated the indirect effects of childhood abuse experiences and SSS on presenteeism. The coefficient of determination (*R*^2^) of presenteeism of this model was 0.177.

## Discussion

### Summary of the results

In this study, we hypothesized that childhood abuse indirectly affects presenteeism by affecting SSS and trait anxiety, and tested our hypothesis by path analysis. We found that SSS and trait anxiety mediate the effects of childhood abuse on presenteeism. The existence of these mediators explains why childhood experiences affect the current status of presenteeism. Moreover, we found that the effects of SSS on presenteeism are completely mediated by trait anxiety. This finding is also novel, and to our knowledge, this is the first report on this issue.

### Childhood abuse and presenteeism

To date, little is known about the association between harmful childhood experiences and the presenteeism of adult workers. Consistent with the results of this study, a previous study reported that people with experiences of childhood abuse have more presenteeism (De Venter et al., 2020). In addition, they reported that the effects of childhood abuse on presenteeism are mediated by the coexistence of current depressive and anxiety disorders. On the other hand, it has also been reported that neuroticism has a mediating effect on the effect of childhood victimization experience on presenteeism (Hashimoto et al., 2022). These previous studies and our present study demonstrate that harmful childhood experiences have a long-term indirect effect on presenteeism in the workplace in adulthood, and that this effect is mediated by anxiety tendencies. Moreover, a strength of the present study is that we clarified that SSS contributes to the indirect effect of childhood abuse on presenteeism, with a strong association with trait anxiety. SSS has been gaining attention recently because it is epidemiologically associated with many mental diseases (Scott et al., 2014). Our results indicate that it is crucial to raise public awareness of the seriousness of childhood abuse, owing to its lasting impact on later life. Preventions and intervention programs targeting childhood abuse are hence recommended. Intervention should be performed at an early stage, such as by strengthening the cooperation between child consultation centers and psychiatric care institutions.

### Trait anxiety and presenteeism

Trait anxiety leads to the development of anxiety disorders and depression (Weger and Sandi, 2018). Trait anxiety is biologically based on genetic or hypothalamic–pituitary axis variations, causing overreactions to stressful life events, and is associated with cognitive behavioral disorders. High trait anxiety also causes insomnia, inadequate attention control, cognitive inhibition, and difficulty in switching tasks (Ansari et al., 2008; Weger and Sandi, 2018). Presenteeism is caused by mental health problems, musculoskeletal disorders, gastrointestinal symptoms, and so on (Kinman, 2019). Occupational stress, fully remote work, sleep disturbances, irregular diets, financial factors, work culture, and the attitudes toward work can also enhance presenteeism (Kinman, 2019; Furuichi et al., 2020; Taya et al., 2020; Hayashida et al., 2021; Shimura et al., 2021, 2022). Of these factors, depression, anxiety, and sleep disturbance are strongly associated with trait anxiety (LeBlanc et al., 2009; Sandi and Richter-Levin, 2009; Nakajima et al., 2014; Uchida et al., 2018; De Venter et al., 2020; Deguchi et al., 2021), and some studies have clarified the association between trait anxiety and presenteeism (Laing and Jones, 2016; Dieguez, 2022). The results of our present study that trait anxiety and presenteeism are strongly associated is consistent with the previous reports. The influence of childhood abuse and SSS can explain part of the mechanism of the association between trait anxiety and presenteeism.

### Clinical significance

The results of this study show that childhood abuse, trait anxiety, and SSS are personal background factors affecting presenteeism in the workplace, and that these factors increase presenteeism. Preventing childhood adverse experiences, such as childhood abuse, leads to the prevention of mental illnesses, such as depression and workplace presenteeism. However, even in patients who experienced adversities in childhood, if clinical evaluation can identify the presence of trait anxiety and low SSS as mediators as suggested by this study, intervention of these factors can be expected to prevent presenteeism. Early intervention of presenteeism is considered to be of clinical significance from the perspective of preventing productivity decline in the workplace. The early detection and intervention of childhood abuse have important implications for personal health and workplace productivity. For example, increasing the cooperation between child guidance centers and psychiatric care institutions, and institutions providing psychological care to abused children, may prevent future adverse events. The results obtained in this study also suggest that childhood abuse, trait anxiety, and SSS are factors that should be considered when elucidating the mechanism of presenteeism and developing countermeasures.

### Limitations

First, as this study used self-administered questionnaires that rely on the subjects’ memories of past experiences, there may be recall bias. Second, as the study was aimed at Japanese adult volunteers, there is a limit to its application to patients with mental illness. Third, this study had a cross-sectional and retrospective design. It is hence necessary to conduct a long-term, large-scale prospective study in the future, to conclude causal associations among these factors.

## Conclusion

The results of our study suggest that the combination of SSS and trait anxiety mediates the association between childhood abuse and presenteeism. Therefore, assessing childhood abuse, SSS, and trait anxiety may help to elucidate the mechanism of workplace presenteeism and to develop measures against presenteeism.

## Data availability statement

The raw data supporting the conclusions of this article will be made available by the authors, without undue reservation.

## Ethics statement

The studies involving human participants were reviewed and approved by Ethics Committee of Tokyo Medical University. The patients/participants provided their written informed consent to participate in this study.

## Author contributions

RN, AS, JM, YIw, AD, YIs, YT, YF, HT, and TI made a significant contribution to the work reported, either in the conception, study design, execution, acquisition of data, analysis and interpretation, or in all these areas; took part in drafting, revising, or critically reviewing the article; gave final approval of the version to be published; have agreed on the journal to which the article has been submitted; and agree to be accountable for all aspects of the work.

## Funding

This work was partly supported by a Grant-in-Aid for Scientific Research (no. 21K07510 to TI) from the Ministry of Education, Culture, Sports, Science and Technology-Japan.

## Conflict of interest

JM has received personal compensation from Otsuka Pharmaceutical, Eli Lilly, Astellas, and Meiji Yasuda Mental Health Foundation, and grants from Pfizer. AS has received personal compensation from Sumitomo Pharma and Eisai. YF has received research and grant support from Novartis Pharma, Otsuka Pharmaceutical, Astellas, Sumitomo Pharma, and Shionogi. TI has received personal compensation from Mochida Pharmaceutical, Takeda Pharmaceutical, Eli Lilly, Janssen Pharmaceutical, MSD, Taisho Toyama Pharmaceutical, Yoshitomiyakuhin, and Daiichi Sankyo; grants from Shionogi, Astellas, Tsumura, and Eisai; and grants and personal compensation from Otsuka Pharmaceutical, Sumitomo Pharma, Mitsubishi Tanabe Pharma, Kyowa Pharmaceutical Industry, Pfizer, Novartis Pharma, and Meiji Seika Pharma; and is a member of the advisory boards of Pfizer, Novartis Pharma, and Mitsubishi Tanabe Pharma.

The remaining authors declare that the research was conducted in the absence of any commercial or financial relationships that could be construed as a potential conflict of interest.

## Publisher’s note

All claims expressed in this article are solely those of the authors and do not necessarily represent those of their affiliated organizations, or those of the publisher, the editors and the reviewers. Any product that may be evaluated in this article, or claim that may be made by its manufacturer, is not guaranteed or endorsed by the publisher.
